# Mental healthcare needs and opportunities in Guatemala

**DOI:** 10.1192/bji.2025.10050

**Published:** 2026-08

**Authors:** Anne Aboaja, Steven McFarlane, Juliet Raphael, Mohamed Ali, Pablo Antonio Cifuentes-Gramajo

**Affiliations:** 1 Forensic Service, Tees, Esk and Wear Valleys NHS Foundation Trust, Middlesbrough, UK; 2 Forensic Service, Tees, Esk and Wear Valleys NHS Foundation Trust, Middlesbrough, UK; 3 Northcroft, University Hospitals Birmingham NHS Foundation Trust, Birmingham, UK; 4 Faculty of Medicine, Universidad de Chile, Santiago, Chile

**Keywords:** Mental health services, Latin America, indigenous health, workforce, access

## Abstract

Consistent with many countries in the region, the Republic of Guatemala likely has a high level of mental health need. However, with high poverty rates and workforce deficits, Guatemala faces challenges in providing accessible mental healthcare across the nation. We describe examples of interventions that have been developed to reduce this mental health gap by addressing the existing barriers to accessing mental healthcare. Within this country profile, we identify further opportunities, such as future mental health legislation, to improve access to services across the human lifespan, especially for at-risk and underserved communities.

This paper aims to provide a mental health country profile of Guatemala, beginning with a contextualised report of the mental health needs of the population, followed by a description of the existing health system services. The barriers to accessing services experienced by underserved and at-risk communities are noted before discussing opportunities to close the mental health treatment gap. References to support this paper were obtained from non-systematic searches of PsycInfo (American Psychological Association, Washington DC, https://www.apa.org/pubs/databases/psycinfo/index), Medline (Ovid) (U.S. National Library of Medicine Bethesda, Maryland, https://www.wolterskluwer.com/en/solutions/ovid/ovid-medline-901), LILACS (Latin American and Caribbean System on Health Sciences Information, https://lilacs.bvsalud.org/en/) and SciELO (https://www.scielo.br/) databases, and Google Scholar. We hand-searched the grey literature, selecting current and relevant information from authoritative sources. We also drew upon the expertise of a consultant psychiatrist in Guatemala named Dr Miguel De León Cardoza.

## Country profile

Following 2 decades of rapid growth, the population of the Republic of Guatemala is 18.4 million.^1^ While Spanish is the official language, many indigenous people speak only 1 of the country’s 24 non-Spanish languages.^2^ Half of the population experience poverty (i.e., living on less than US$2.15 a day), with the highest rates among the indigenous and rural communities. Equally high levels of food insecurity and digital exclusion are reported.^1^

## Mental health of the general population

A nationwide household survey estimated 1 in 4 (27.8%) of the adult population have a mental disorder. This includes anxiety disorders (20.6%), mood disorders (8.0%), substance misuse, (7.8%; 5.2% alcohol) and non-affective psychotic disorders (8.0%).^3^ Limitations of the survey include the exclusion of under 18s and over 65s and the absence of data on personality and developmental disorders.

Indigenous adults are particularly vulnerable to living with mental disorders.^3^ Whether this reflects their higher rates of poverty and rural living or not, research indicates relatively higher mental health service investments are needed in this high-risk population.

The Global Burden of Disease Study showed the suicide mortality rate in Guatemala increased by 27.9% between 1990 and 2019, and, consistent with Latin America, the rate is higher in males (9.2 suicides per 100 000) than females (2.4 per 100 000).^4^ Compared to older adults, Guatemalans aged 25 to 49 are disproportionately affected by suicide.^4^ Although research to understand and prevent suicide in this age range is limited, studies have been conducted among people approaching this high-risk age. Secondary analysis of Global School Health Survey data found amongst 4274 adolescents in Guatemala, the 12-month prevalence of self-reported attempted suicide was 20.2% for girls and 12.2% for boys. The finding that loneliness was a significant risk for attempting suicide can inform the development of targeted interventions in this group.^5^

## Mental healthcare system

### Regulation and finance

Seeking to meet the health needs described above, Guatemala has a stand-alone national mental health plan which partially meets human rights standards.^6^ Although under discussion, at the time of writing, there is no dedicated mental health legislation; the care and protection of persons with mental disorders falls within generic health and disability legislation. The overall healthcare budget, which is 2.3% of the gross domestic product (GDP),^2^ leaves only a small allocation for mental health services.^1^

### Provision

In-patient mental healthcare is provided through one mental health hospital, a psychiatric unit within a general hospital and one forensic psychiatry unit. No formal arrangements currently exist for the provision of mental healthcare in prisons, although the proposed mental health legislation calls for reforms to create dedicated mental health centres inside prisons. There is a disproportionately high number of out-patient facilities in Guatemala City, with relatively few in rural areas and regions with high indigenous populations.^7^

Services are delivered mainly by psychologists (46.15 per 100 000 population).^6^ In comparison, the number of psychiatrists and mental health nurses is extremely low (0.49 and 1.07 per 100 000 population, respectively).^6^ The number of psychiatrists working specifically with children and adolescents is even lower (0.04 per 100 000 population).^6^

## Barriers to accessing mental health systems and opportunities to improve access

The national household survey found that of those with a diagnosable mental disorder, only 2.3% had consulted a psychiatrist or other medical doctor for their emotional or mental health problems, while 2.5% sought help from a religious guide.^3^ A combination of social, economic and workforce factors act as barriers to accessing mental healthcare and treatment, particularly for indigenous communities.

### Social factors

Non-Spanish monolingualism among some indigenous groups presents a challenge to accessing mental health services delivered by professionals who speak only Spanish.^2^ Effective access to mental health services will be improved by working with indigenous communities to reorient the service delivery process and structure to the community; for example, ensuring healthcare professionals can adapt to and communicate in the patient’s language; including religious guides as supplementary mental healthcare providers; acknowledging different understandings of mental health; translating medicines information leaflets into all 25 languages; and providing opportunities for members of the indigenous community to train as psychiatrists and mental health nurses.^8^

Among the most impoverished communities, digital exclusion remains a barrier to accessing online mental health information. However, where Wi-Fi is available to underserved communities, a social media-based health campaign has been used to disseminate cognitive–behavioural therapy (CBT)- and dialectical behaviour therapy (DBT)-based psychoeducation to almost 85 000 individuals.^9^ Having demonstrated feasibility, research is now needed to test the effectiveness of this intervention.

### Economic

Poverty is a key barrier to accessing mental services in Guatemala. Primary care staff managing common mental disorders among rural indigenous communities report a lack of local specialist mental health services and describe the logistical and financial challenges of low-income patients travelling long distances to receive care at the national psychiatric hospital.^6,7,10^ In a country where 29% of the daily minimum wage is required to purchase a daily dose of an antipsychotic and 17% is required for an antidepressant, the cost of psychotropic medication is another barrier to receiving mental health treatment.^1,7^

According to a systematic review of poverty and mental health in low- and middle-income countries, food insecurity has a stronger association with mental health outcomes, compared to other poverty markers such as family income,^11^ yet it is highly plausible that increased family income would improve mental healthcare access. The complex interplay of socioeconomic factors, the prevalence of mental health conditions and limited access to services points to policy-level solutions to address the wider determinants of mental health as well as the implementation of affordable universal mental health coverage as mentioned in the proposed mental health legislation.^12^

### Workforce

A barrier to accessing mental healthcare is the lack of mental health nurses and psychiatrists, especially in rural communities. Globally, similar shortages have been addressed in higher- and lower-income countries through: (a) integrating mental healthcare in primary care systems, (b) task-shifting or sharing, such as redistributing mental health-related tasks to trained non-specialists, including lay people, (c) telepsychiatry and (d) implementing workforce retention strategies.^13^

In Guatemala, a mental health curriculum developed to build capacity and improve access to mental healthcare in rural areas was used to train primary care physicians to offer brief alcohol interventions using motivational interviewing.^14^ In response to the elevated suicide risk among young people,^6^ task-shifting schoolteachers in Guatemala have been trained to identify at-risk students, undertake assessments and provide community-level interventions.^15^ Serving as a link between the lay community and mental health workers, these teachers also facilitate young people and their parents accessing specialist care.^15^ Teachers who participated in the study found training acceptable and reported increased knowledge; however, a larger-scale, controlled evaluation is required to demonstrate overall effectiveness in increasing knowledge and achieving benefits for young people over time, before the intervention can be scaled up.

Effective strategies used to retain doctors in other countries could protect against shrinkage of the existing workforce. For example, providing educational opportunities to collaborate with academics alongside clinical work and awarding financial benefits for those who work in rural and other underserved areas.^13^

## Conclusions

There is a clear need for new large-scale epidemiological studies and longitudinal research, without which it is difficult to estimate the latest mental health need and trends and to understand how and why mental health risks change over time. This information is crucial for accurate resource allocation and effective policy development and service design. Available data suggests that the existing mental health system lacks the policies, funding and workforce to address the needs of the nation through the provision of accessible services. The proposed mental health legislation promises to address some of the existing barriers to accessing mental healthcare and will strengthen national policy-level programmes which are required to improve access, particularly for those in underserved and at-risk communities. However, effective universal mental health coverage will not be achieved without full cultural transformation of mental health services. Given the cultural diversity of the country and the available evidence on the mental health needs of indigenous people and their experience of existing mental health services, it is recommended policymakers encourage mental health service providers in adopting and auditing the implementation of a framework for cultural adaptation.^8^ While a small but growing range of mental health interventions have been developed and tested to meet the specific needs of local populations, further evaluation is required to demonstrate effectiveness before scaling-up can be recommended.
